# The Abdominal Aortic Aneurysm and Intraluminal Thrombus: Current Concepts of Development and Treatment

**DOI:** 10.3389/fcvm.2015.00019

**Published:** 2015-05-26

**Authors:** Aleksandra Piechota-Polanczyk, Alicja Jozkowicz, Witold Nowak, Wolf Eilenberg, Christoph Neumayer, Tadeusz Malinski, Ihor Huk, Christine Brostjan

**Affiliations:** ^1^Department of Surgery, Medical University of Vienna, Vienna, Austria; ^2^Department of Biochemistry, Medical University of Lodz, Lodz, Poland; ^3^Department of Medical Biotechnology, Jagiellonian University, Krakow, Poland; ^4^Department of Chemistry and Biochemistry, Ohio University, Athens, OH, USA

**Keywords:** aortic aneurysm, abdominal, intraluminal thrombus in aortic aneurysms, neutrophils, heme oxygenase-1, nitric oxide synthase

## Abstract

The pathogenesis of the abdominal aortic aneurysm (AAA) shows several hallmarks of atherosclerotic and atherothrombotic disease, but comprises an additional, predominant feature of proteolysis resulting in the degradation and destabilization of the aortic wall. This review aims to summarize the current knowledge on AAA development, involving the accumulation of neutrophils in the intraluminal thrombus and their central role in creating an oxidative and proteolytic environment. Particular focus is placed on the controversial role of heme oxygenase 1/carbon monoxide and nitric oxide synthase/peroxynitrite, which may exert both protective and damaging effects in the development of the aneurysm. Treatment indications as well as surgical and pharmacological options for AAA therapy are discussed in light of recent reports.

## Epidemiology and Risk Factors

**Figure 1:**
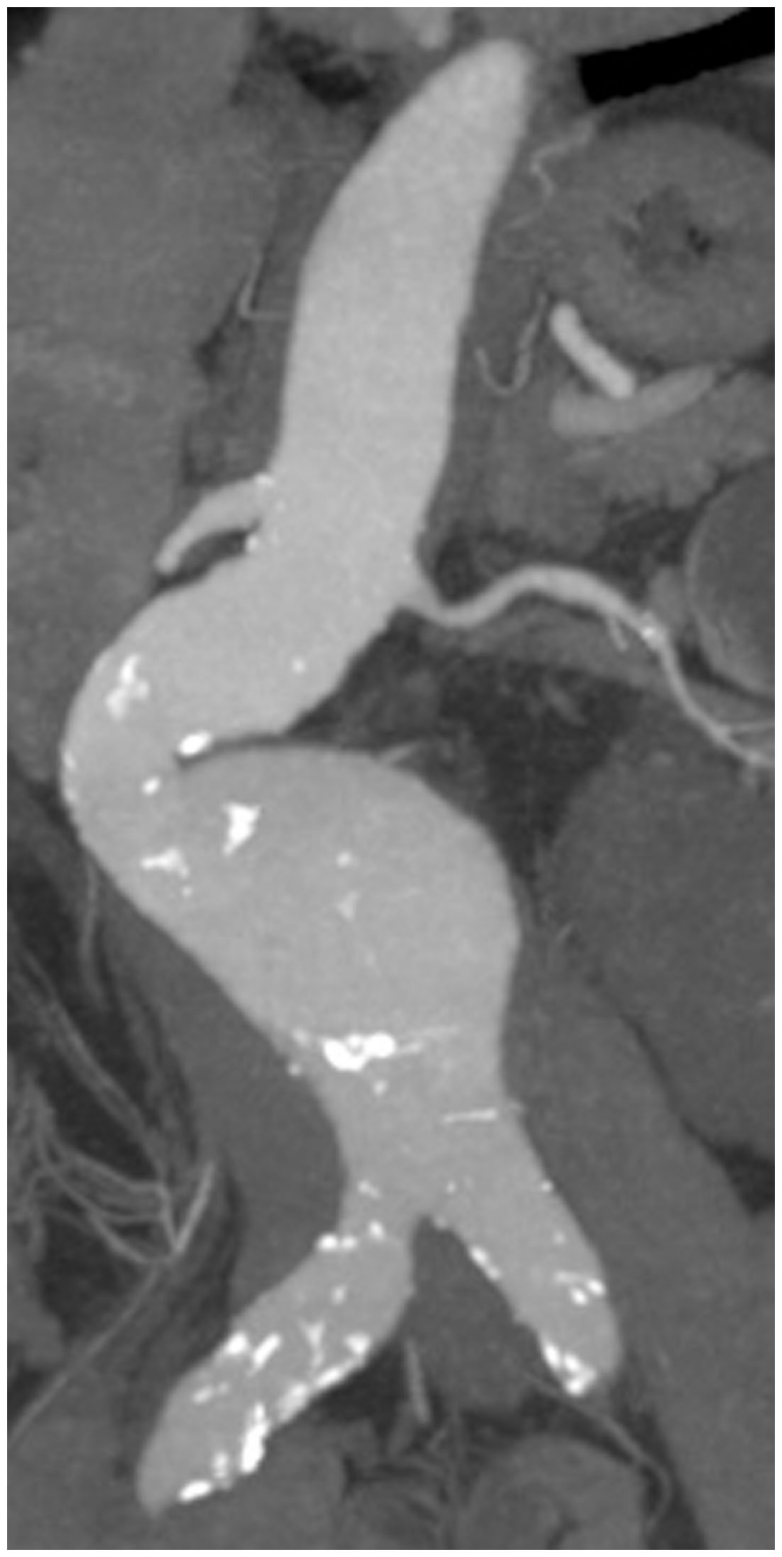
**CT recording of an infrarenal AAA with eccentric shape and sites of calcification (atherosclerotic plaques highlighted in white)**.

“Aneurysm” is defined as dilatation of an artery being at least 1.5 times larger than its expected normal diameter (1). Thus, an abdominal aortic aneurysm (AAA) is given when the maximum diameter is 30 mm or more (2). Approximately, 80% of AAAs occur in the infrarenal aorta (3). In general, “atherosclerotic” aneurysms represent the vast majority of AAAs (Figure 1).

The prevalence of AAAs depends on patients’ age, gender, and geographical location (4). Primarily, elder men are affected. Men’s prevalence of AAAs below 50 mm in diameter increases from 1.3% below 45 years to 12.5% above 75 years (2). For women, the prevalence ranges from 0 to 5.2%, respectively, but the risk of rupture is four times greater. The underlying mechanisms of sex differences are not fully understood, although women seem to be protected by female sex hormones (2). Smoking is another strong risk factor for the development of AAAs due to its promoting effects on inflammation, proteolysis, and smooth muscle cell (SMC) apoptosis (5). Enhanced aneurysm growth and an increased risk of rupture have been described (6). Other risk factors comprise previous vascular aneurysms, coronary artery and cerebrovascular disease, atherosclerosis, hyperlipidemia, and hypertension (4). An observational study recently demonstrated that a low vitamin D status was associated with the presence of larger AAAs in elder men (7). Moreover, various microorganisms have been associated with the pathogenesis of AAAs (8).

In addition to these environmental components, genetic aspects play an important role. A positive family history for AAA especially in male first-degree relatives is associated with an increased risk for AAA (9). Moreover, alterations on chromosome 9p21 are accompanied with a 20% increased risk of developing AAA (10). Other genetic approaches suggested that aberrations of lipid metabolism and proteolytic pathways are the key contributors to disease. Some of these associations (e.g., lipoprotein receptor-related protein-1) are not associated with atherosclerosis, indicating pathways unique to AAA (11). Distinct connective tissue disorders such as Marfan syndrome, Ehlers–Danlos syndrome, and Loeys–Dietz syndrome also go along with an increased risk for AAAs (12).

## The Intraluminal Thrombus

**Figure 2:**
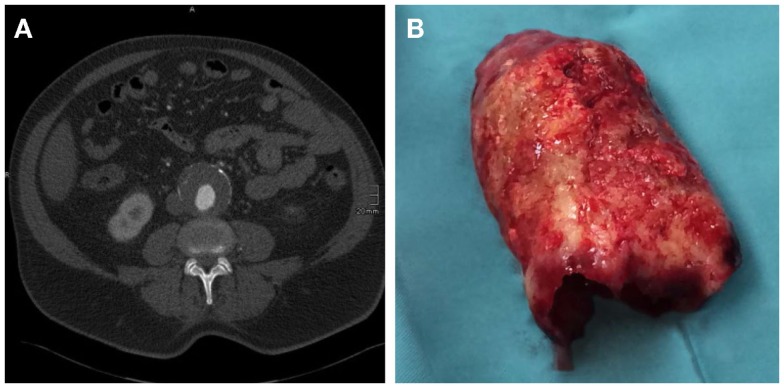
**(A)** CT axial image of an infrarenal AAA with intraluminal thrombus. **(B)** Corresponding, massive, intraluminal thrombus removed during open surgical repair.

In 70–80% of AAA patients, the vessel wall is covered by an intraluminal thrombus (ILT, Figure 2), which generally does not preclude blood flow and shows little compression throughout the cardiac cycle (13, 14). While mural thrombosis is frequently observed in aneurysmal disease, the complete vessel occlusion is a comparably rare event associated with a high rate of mortality (15).

An inhomogeneous thrombus structure was reported early on, with a stiffer luminal side composed of a dense fibrin network invaded by leukocytes and erythrocytes (16). By contrast, the medial and abluminal ILT layers were found to be largely devoid of cellular constituents and to exhibit progressive fibrinolysis and hence decreased thrombus strength and stiffness (17). Recent reports have indicated that ILT structure may indeed be more diverse than previously thought. When luminal, medial, and abluminal ILT layers were evaluated for mechanical properties, three subtypes of ILTs were identified (13). While the majority of investigated thrombi displayed a multi-layered morphology with gradually decreasing strength and stiffness from the distinct luminal to the thick medial/abluminal side (type 1), examples of multi-layered ILTs with an abrupt loss of mechanical resistance from luminal layers to rather thin and highly degraded medial/abluminal layers were also observed (type 2). Of interest, a third type of single-layered ILT of almost fluid-like consistency was reported for a limited number of cases (13). Future studies will have to elucidate the pathogenesis and cellular/molecular composition of these distinct ILT variants. Importantly, based on the possibilities of medical imaging, the potential correlation of ILT subtype with disease progression and risk of rupture should be evaluated.

Macromolecular transport is promoted by centrifugal convection from the luminal to the abluminal side of the fibrin network and is further supported by the system of so-called “canaliculi” (17). Thus, molecules released and activated within the thrombus are readily transported to the vessel wall and affect aneurysm growth. Animal models have shown that limiting thrombus development with inhibitors of platelet activation strongly suppresses aneurysm formation (18). The effect is accompanied by a reduction in leukocyte infiltration and lower release of proteases within the mural thrombus, which results in decreased degradation of elastic fibers, and promotes thrombus colonization by SMCs. Thus, by sequestering and activating platelets, erythrocytes, neutrophils, and macrophages, the ILT exposes the vessel wall to a local milieu of highly concentrated cytokines, proteases, and reactive oxygen species (ROS) that promotes aneurysm development. In line, clinical studies report that ILT thickness correlates with AAA diameter, matrix metalloproteinase (MMP) levels, elastin degradation, and SMC apoptosis (19, 20). Furthermore, the presence of a large thrombus leads to localized hypoxia at the underlying aortic wall, which triggers adventitial angiogenesis and aggravates inflammatory infiltration from the outer vessel layers (21).

Despite this role in supporting the inflammatory and proteolytic mechanisms of AAA pathogenesis, the mural thrombus has repeatedly been suggested to protect the aneurysm from rupture by reducing the peak wall stress and altering wall stress distribution (22). However, this protective role seems to depend on the degree of thrombus attachment to the vessel wall (23). When bearing in mind that large thrombi are reportedly associated with faster AAA growth, the proteolytic weakening of the AAA wall may be the more predominant ILT effect when compared to wall stress relief (24).

Proteomics analysis of proteins sequestered by the ILT fibrin network revealed the expected prevalence of platelet-derived proteins such as clusterin and thrombospondin-1 (25), which are likely to be subjected to proteolytic processing within the local thrombus milieu thereby altering or activating protein function (26). Furthermore, as the ILT constitutes a site of continuous hemostasis and fibrin destruction, the respective coagulation and fibrinolysis factors are detected in thrombus tissue and elevated in AAA patients’ blood (27). Fibrinogen, D-dimer (a cross-linked fibrin degradation product) and TAT (thrombin–antithrombin complex) prevailed in meta-analysis as diagnostic markers (28). Furthermore, circulating concentrations of hemostatic and fibrinolytic markers correlated with AAA and ILT size (29) and D-dimer blood levels were suited to predict the AAA growth rate (30). Importantly, large, population-based health screenings identified elevated blood levels of fibrinogen and tissue plasminogen activator to be associated with the occurrence of AAA within the subsequent 10–20 years follow-up period. The fact that these plasma proteins were elevated years before the clinical manifestation of disease further supports the notion that deregulated hemostasis/fibrinolysis is intricately involved in AAA pathogenesis – starting at an early stage of disease (31).

The factors potentially protecting or predisposing an individual to AAA development have been subjected to intense investigation. Loss of endothelial homeostasis resulting in pro-inflammatory and pro-coagulant activation is proposed to trigger the onset of disease. Two molecules that are central in regulating endothelial homeostasis and may thus exert a protective function are carbon monoxide (CO) and nitric oxide (NO) (32, 33). However, a more complex picture has emerged with CO, NO, and their enzymatic or reactive by-products exhibiting additional effects on, e.g., SMC proliferation and survival, which may promote rather than inhibit AAA development (34, 35). The loss of SMCs is a hallmark of the weakening vessel wall. In addition, AAA pathogenesis involves the destruction of elastic fibers and other matrix components, which seems to be vitally regulated by the accumulation of neutrophils in the growing ILT (36). These central mechanisms of disease development are highlighted in the following review sections.

## Role of Heme Oxygenase 1 in AAA and ILT

**Figure 3:**
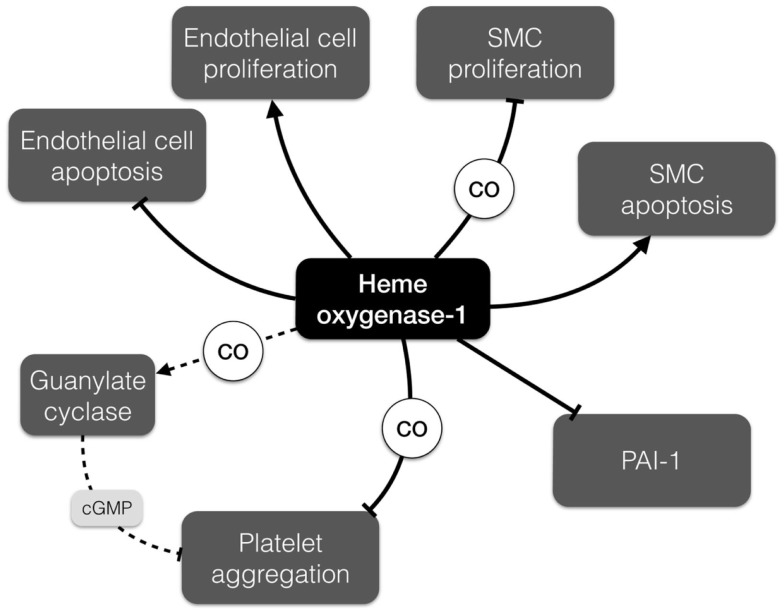
**Differential effects of heme oxygenase-1 (Hmox1) relevant to AAA development**. Hmox1 promotes endothelial cell proliferation and inhibits their apoptosis, but has opposite impact on the proliferation and apoptosis of smooth muscle cells. It can also modulate thrombus formation by inhibition of platelet aggregation and promotion of fibrinolysis. cGMP, cyclic guanosine monophosphate; PAI-1, plasminogen activator inhibitor-1; SMC, smooth muscle cell.

Heme oxygenase-1 (Hmox1) and products of its activity can affect blood coagulation and formation of the thrombus. Hmox1 is an enzyme degrading heme – as released by erythrocyte trapping and hemagglutination in the thrombus – to carbon monoxide (CO), ferrous iron (Fe^2+^), and biliverdin, which is subsequently reduced by biliverdin reductase to bilirubin. Hmox1 has anti-oxidant, anti-inflammatory, and cytoprotective activity that is crucial for blood vessel homeostasis (32) (Figure 3). Carbon monoxide activates guanylate cyclase, increases the level of cGMP (cyclic guanosine monophosphate), and subsequently inhibits platelet aggregation (37). Moreover, CO has been shown to suppress plasminogen activator inhibitor-1 (PAI-1) in a lung ischemia-driven thrombosis model (38) and subsequently affects fibrinolysis. Accordingly, both CO and bilirubin suppress PAI-1 in Hmox1-deficient mouse embryonic cells (39).

The majority of studies characterizing the role of Hmox1 in hemostasis and thrombosis have been performed on the occlusive types of thrombosis as opposed to AAA. Lack of heme oxygenase-1 in *Hmox1^−/−^* mice leads to the faster occurrence of an occlusive thrombus in the carotid artery after photochemical injury (40). Basal levels of carotid tissue factor, platelet counts, bleeding time, and prothrombin time were not different in *Hmox1*^+^*^/^*^+^ and *Hmox1^−/−^* mice. However, vascular injury induced higher levels of arterial tissue factor and von Willebrand factor in *Hmox1^−/−^* than in *Hmox1*^+^*^/^*^+^ mice. Also, photochemical injury led to the endothelial damage only in *Hmox1^−/−^* mice. Finally, *Hmox1*^+^*^/^*^+^ mice transplanted with *Hmox1^−/−^* bone marrow showed accelerated thrombosis in comparison to the *Hmox1*^+^*^/^*^+^ mice that received *Hmox1*^+^*^/^*^+^ bone marrow. Interestingly, faster occlusive thrombus formation in *Hmox1^−/−^* mice could be rescued with biliverdin, or when mice were inhaled with sublethal doses of CO (40).

Similarly, in the model of stasis-induced thrombosis in the *inferior vena cava* (IVC), the clot was bigger in *Hmox1^−/−^* mice (41). IVC ligation increased *Hmox1* transcription and protein levels in wild type endothelial and SMCs as well as in infiltrating cells. It was demonstrated that IVC ligation in *Hmox1^−/−^* mice induced a higher activation of nuclear factor kappa B (NF-κB) transcription factor and increased the inflammatory response as reflected by the expression of interleukin-6 (IL-6), monocyte chemoattracting protein-1 (MCP-1), stromal cell-derived factor-1, and KC (the murine homolog of interleukin-8) than in wild type animals. Importantly, the activity and expression of MMP-9 were also elevated in *Hmox1^−/−^* mice. Finally, similar to the model of carotid artery injury in the work by True et al. (40), the *vena cava* ligation led to the increased production of tissue factor in *Hmox1^−/−^* mice (41).

Cobalt protoporphyrin IX (CoPP), a known inducer of heme oxygenase-1, inhibits formation of the thrombus in response to laser ablation of endothelium in cremaster arterioles, whereas tin protoporphyrin IX (SnPP), a heme oxygenase-1 inhibitor, leads to enhanced thrombus formation (42). Interestingly, in a murine model of aorta allotransplantation, the thrombus was formed when aortas from *Hmox1^−/−^* were grafted (43). The effect of the lack of *Hmox1* was rescued with carbon monoxide releasing molecule-2 (CORM-2) (43). Moreover, administration of hemin, which not only induces Hmox1 but also promotes oxidative stress, resulted in faster clot formation in response to ferric chloride in *Hmox1^−/−^* mice than in *Hmox1*^+^*^/^*^+^ mice, which was not observed under basal conditions (44). Noteworthy, in animals with a normal level of *Hmox1* hemin may have a protective activity. Prophylactic treatment of Wistar rats with hemin reduced carotid thrombus formation in response to the electric stimulation (45). A similar observation was found in the mouse cremaster microvascular circulation, where hemin delayed formation of the thrombus in response to ferric chloride (46).

Despite the fact that the majority of functional studies regarding the role of Hmox1 in thrombus formation were conducted in the context of occlusive thrombosis, there are several findings that implicate Hmox1 in AAA pathobiology. Of note, expression of *Hmox1* is increased in rat aorta on days 7 and 10 after AAA induction with elastase (47). Enhanced expression of *Hmox1* prevents endothelial cell apoptosis and facilitates endothelial proliferation (32). By contrast, upregulation of Hmox1 in vascular SMCs induces p53 expression and promotes apoptosis (48). Noteworthy, increased SMC death and a high level of p53 is a common feature of AAA lesions and the weakening vessel wall (49). Furthermore, carbon monoxide inhibits the rat aortic SMC proliferation under hypoxic conditions in response to endothelin-1 (34). Moreover, probucol, which is used to prevent restenosis, increases Hmox1 levels in SMCs and therefore inhibits their proliferation (50). The diverse effects that Hmox1 and its enzymatic products may exert in the AAA setting (such as reducing thrombus formation, yet increasing SMC apoptosis) are summarized in Figure 3.

Importantly, the level of *Hmox1* expression in humans is modulated with the microsatellite polymorphism of the gene promoter (51). Namely, a longer promoter with more guanidine–thymidine (GT) repeats (*n* ≥ 29) results in lower basal expression of *Hmox1* and weaker upregulation in response to stimuli (52). It was shown that human umbilical vein endothelial cells with a short *Hmox1* promoter (*n* ≤ 23) survive better under oxidative stress, proliferate more effectively in response to vascular endothelial growth factor and produce less pro-inflammatory cytokines such as IL-1β, IL-6, and soluble intercellular adhesion molecule-1 (52). The frequency of carriers of the short GT repeat allele of the *Hmox1* promoter was significantly lower in Austrian AAA patients than in coronary or peripheral artery disease-matched controls (53). Similarly, there was a higher frequency of the longer GT repeat allele of the *Hmox1* promoter in patients with cerebral aneurysms (54). This may suggest that a higher expression or inducibility of *Hmox1* may play a protective role against AAA development. By contrast, in a group of Croatian AAA patients, there was a higher frequency of the carriers of short GT repeats in the *Hmox1* promoter than in the non-AAA group (55). Thus, the relation between AAA development and *Hmox1* promoter polymorphism requires further analysis.

## Role of NO and Nitroxidative Stress in Aortic Aneurysm Formation

**Figure 4:**
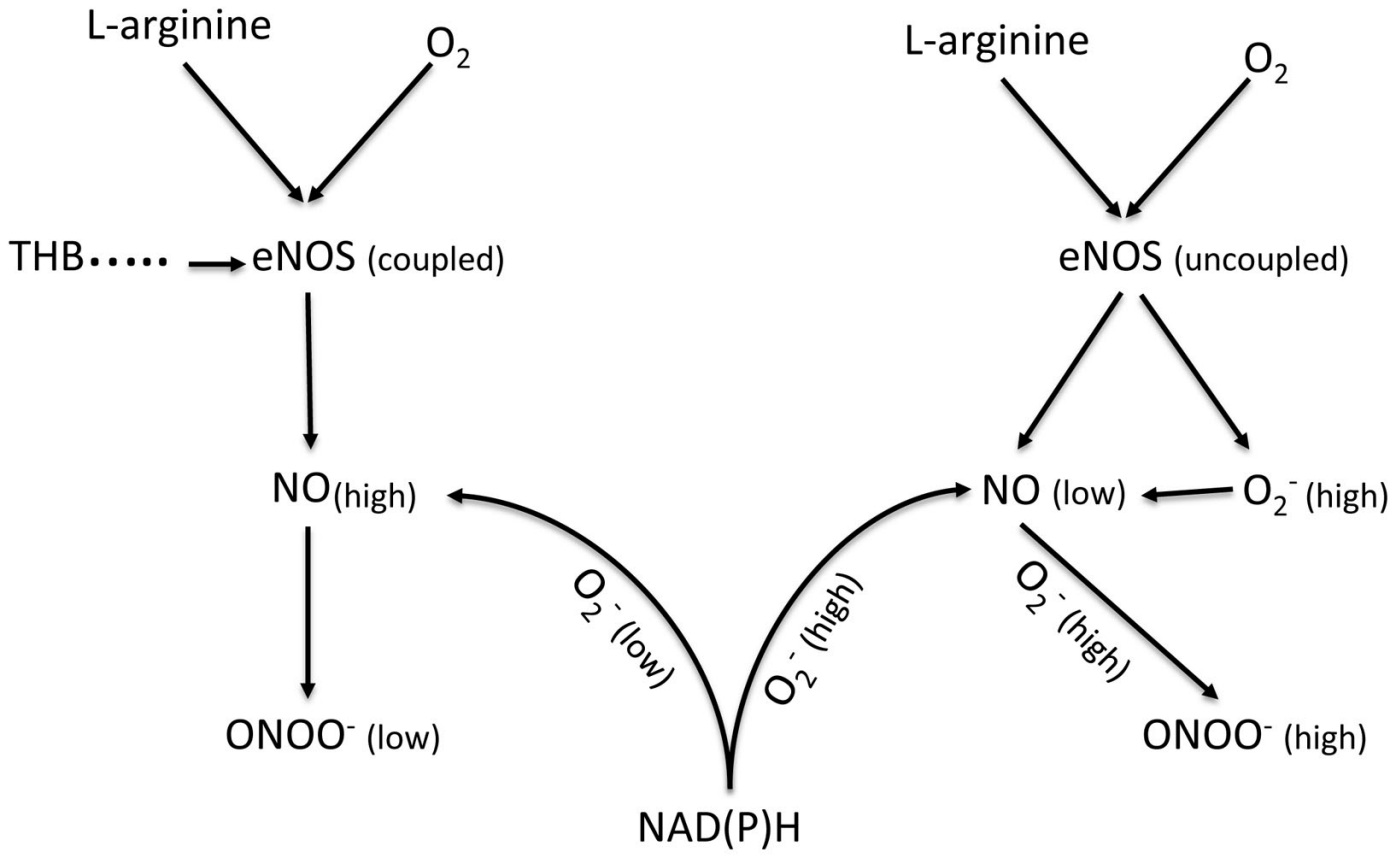
**Schematic diagram showing the generation of NO and ONOO^−^ from a coupled eNOS and an uncoupled eNOS in a normal and a dysfunctional endothelium, respectively**. eNOS, endothelial nitric oxide synthase; NAD(P)H, nicotinamide adenine dinucleotide (phosphate); TDB, tetrahydrobiopterin.

Nitric oxide (NO) can be produced by three nitric oxide synthases (NOS): endothelial – eNOS, neuronal – nNOS and inducible – iNOS. In endothelium, NO is produced mainly by eNOS (Figure 4). Its release depends on the velocity of blood flow and the diameter of the vessel. In the cardiovascular system, NO regulates the blood flow as well as prevents platelet and leukocyte adhesion and aggregation (33, 56, 57). Under laminar flow, there is a homogeneous thin layer (about 1–3 μm) of NO in close proximity to the arterial wall. However, under turbulent or semi-turbulent flow, the NO layer can be depleted (58). Therefore, NO production from the endothelium under turbulent flow is significantly higher than under laminar flow. Under this turbulent flow and under intensive production of NO, eNOS can become uncoupled, which leads to dysfunction of the endothelial cells (58, 59). This process is observed in many cardiovascular diseases including atherosclerosis, hypertension, stroke, diabetes, and aneurysm. The uncoupling of eNOS can be attributed to a shortage of substrates of eNOS (L-arginine and/or oxygen) as well as cofactors of eNOS like tetrahydrobiopterin (THB) (60, 61). Uncoupled eNOS can concomitantly generate NO and superoxide $\left(O_{2}^{-}\right)$. NO can react rapidly in a diffusion controlled reaction with $O_{2}^{-}$ to produce peroxynitrite (ONOO^−^). Peroxynitrite is a short lived (*t*_1/2_ < 1 s) molecule with an oxidation power that is much higher than that of NO or $O_{2}^{-}$. Therefore, high concentrations of ONOO^−^ can cause significant damage to proteins, enzymes, and DNA in a biological milieu (62, 63).

The role of NO in the development of aneurysm is ambiguous and unclear. There are several studies concerning eNOS- and iNOS-derived NO in the pathology of aneurysms performed on animal models and human beings (64–68). However, the results produced by these studies are very often contradictory. Aneurysmal degeneration is a result of biochemical and biomechanical processes, which finally lead to the partial destruction of the aortic wall (49). Nitric oxide and peroxynitrite may be involved in many of these processes. The effect of turbulent flow will be more pronounced with the increase in diameter and asymmetry of aneurysms (69). Based on our studies, the change of flow from laminar to turbulent would lead to a higher level of NO concentrations and a higher expression of eNOS (62, 63). However, an increase in eNOS expression does not always produce a higher level of bioavailable NO (diffusible NO, which can be involved in cellular signaling), because it is not usually accompanied by an increase in the levels of L-arginine or cofactors like THB. At an insufficient level of substrates and cofactors, the dimeric form of eNOS enzyme can be uncoupled and the net effect of uncoupled eNOS correlates inversely with the level of bioavailable NO and directly with ONOO^−^ concentrations. Thus, while NO production by eNOS is known to play a protective role in the cardiovascular system by its vasodilatory effect, Gao et al. found that eNOS uncoupling/THB deficiency accelerated the formation of AAA in mice (64). Comparably, it has been suggested that eNOS deficiency increased in atherosclerosis in Western-type diet-food apoE knock-out mice and triggered spontaneous aortic aneurysms (66). Furthermore, NO may play a role in the MMP regulation in AAA: NO produced via eNOS inhibits MMP activity and inhibits SMC migration (65, 68).

Animal models demonstrated that inhibition of iNOS decreased NO generation and inhibited aneurysm formation. However, the opposite effect was also reported. Lee et al. demonstrated in animal models that iNOS does not play a requisite role in the process of elastase-induced experimental aneurysmal degeneration in mice (67). The authors also suggested that therapeutic treatment of aneurysms by inhibition of iNOS may have a deleterious effect. Most published studies on models of aneurysm indicate that iNOS expression increases while eNOS decreases during aneurysmal degeneration.

We believe that a likely reason for these confusing results is due to the heterogeneous distribution of NOS along the wall of an aneurysm. In our opinion, the type of flow (laminar versus turbulent) could be a determining factor, which will influence the kinetics of NO release, expression of eNOS and iNOS, and NO bioavailability in different segments of aneurysmal tissue. Therefore, one can assume that it is highly unlikely that a lateral NO distribution in the aneurysm will be homogeneous, but it will not be. Thus, analysis of a sample of aneurysm without information providing specific locations (coordinates) of the aneurysmal tissue may be flawed. An indication of NO production by iNOS has been closely associated with inflammation, which follows an increase in the dysfunction of the endothelium, the increase in eNOS uncoupling, and an increase in peroxynitrite and nitroxidative stress. It is well accepted that ONOO^−^ is directly involved in the triggering of inflammation. Endothelial, bioavailable NO is cytoprotective, while peroxynitrite is cytotoxic. The balance between the concentrations of these two molecules has to be measured along the arterial wall at well-defined coordinates. These kinds of measurements have never been done and this is likely the reason for the very conflicting picture seen for the role of NO in the development of aneurysms. Recently, our laboratory developed a system of nanosensors (diameter of lower than 300 nm), which can be placed near the endothelium of an aortic aneurysm for the simultaneous, *in situ* measurements of NO, ONOO^−^, and $O_{2}^{-}$ (62, 63). The preliminary data obtained from these experiments indicate a substantial difference in the kinetics and concentration of the release of NO, as well as the release of the other components of nitroxidative stress at different segments of aneurysms. NO plays a significant role in the early events in aneurysm formation and this mechanism may not be related to hypertension. During this early event, uncoupled eNOS starts to produce significant concentrations of ONOO^−^, changing the balance between the cytoprotective NO and the cytotoxic ONOO^−^ (70). The NO/ONOO^−^ imbalance stimulates iNOS, which starts to produce uncontrollably high levels of NO, as a protective measure. However, this increase in NO production by iNOS causes further uncoupling of eNOS due to local consumption of L-arginine. The net effect of these processes (nitroxidative stress) stimulates a cascade of events, which lead to the elevated generation of additional oxidative and nitroxidative species, including $O_{2}^{-}$, ONOO^−^, and H_2_O_2_. The stimulation of NAD(P)H, under this condition, contributes to the elevation of $O_{2}^{-}$, oxidation of THB, and the further enhancement of ONOO^−^ levels (62, 64).

Therefore, the low level of bioavailable NO and the high level of nitroxidative and oxidative stress can be considered as important factors in the initial stage of aneurysm development. This process can be similar to that observed in atherosclerosis. Nitroxidative stress can trigger several processes leading to the injuring of endothelial cells and SMCs, upregulation of chemotactic cytokines, upregulation of NAD(P)H and adhesion molecules, as well as activation of MMPs (71). All of these processes can contribute to vessel wall remodeling and breakdown. As the aneurysm develops further, NO is involved in the inhibition of smooth muscle proliferation, nitroxidative stress, and the change of angiogenic activities (35, 72). This may result in the serious destruction of extracellular matrix and elastic fibers. The net effect of NO and ONOO^−^ action can be the thickening and weakening of the arterial wall and finally its rupture. We believe that the ratio of NO concentration to ONOO^−^ concentration and non-homogenous distribution of oxidative/nitroxidative stress plays a crucial role in the development of the aneurysm.

## Role of Neutrophils in Aortic Aneurysm Formation

The bulk amount of ROS and reactive nitrogen species (RNS) in AAA and ILT is produced by activated polymorphonuclear cells like neutrophils. Neutrophils have pro-oxidant activities via, e.g., NADPH oxidase and myeloperoxidase. Myeloperoxidase is a heme enzyme, which is expressed in 95% of polymorphonuclear neutrophils (PMNs). Both myeloperoxidase and NAPDH oxidase are primarily involved in the generation of ROS/RNS (73). Ramos-Mozo et al. showed a decreased catalase activity in circulating PMNs as well as in plasma from AAA patients, indicating that neutrophils of AAA patients have a reduction in anti-oxidant enzymes (74). By contrast, H_2_O_2_ levels and MPO levels in isolated PMNs were significantly higher than in controls. Therefore, a redox imbalance toward increased oxidative stress in AAA patients could be a key factor in AAA formation (74). However, PMNs do not only contribute to oxidative stress but also to proteolytic degradation of the aortic media and to adventitial inflammation (75). Importantly, PMN depletion showed a significant inhibition of experimental AAA formation thus pointing to the central role of neutrophils in AAA pathogenesis (76).

The luminal layer of the ILT is the predominant site of leukocyte retention. In the luminal ILT IL-8, a neutrophil chemotactic factor is released four times higher than in the AAA wall (77). Leukotriene B4 (LTB4) is another potent leukocyte chemoattractant and mediator of inflammation. Houard et al. showed thrombus-derived LTB4 as a mediator of neutrophil chemotaxis (78). Again, the luminal layer of the ILT had the highest activity. Furthermore, the alternative complement pathway was found to be activated in the AAA setting (79) and C5a had the specific ability to chemoattract neutrophils and trigger oxidative burst by inducing the release of CXC chemokines (80). Pagano et al. showed in a murine model that elastase-induced AAA is indeed complement (C3a, C5a) dependent (79). Neutrophils are 12 times more numerous in clots than in blood because these cells have a high affinity for the fibrin–fibronectin network. They bind to fibrin via integrins and to platelet-exposed P-selectin via the expression of the sialyl Lewis-X-containing polysaccharide ligand (81). Neutrophils are terminally differentiated cells, which undergo constitutive apoptosis after binding; this process is postponed upon NF-κB activation in neutrophils (82) as facilitated in the context of the ILT.

The presence of an ILT has been associated with a thinner arterial wall (21), more extensive elastolysis, a lower density of SMCs in the media, and a higher level of immuno-inflammation in the adventitia (19). This suggests that an important part of the protease activity originates from the ILT in contrast to the previously suspected direct generation within the AAA wall. The ILT is particularly rich in pro- and active forms of MMP-9 (83), and MMP-9–lipocalin complexes, which are of neutrophil origin (84). Localization of neutrophils in the luminal part of the thrombus is associated with increased levels of MMP-8, MMP-9, and elastase compared with other (medial and abluminal) layers of the ILT (36). Neutrophils release granular serine proteases such as urokinase plasminogen activator, elastase, proteinase 3, and cathepsins from their azurophilic granules. MMP-9 and MMP-8 are released from gelatinase granules. The cysteine proteases are also potent elastolytic and collagenolytic enzymes associated with AAA development. Several cathepsins (85) and dipeptidyl peptidase I (86) have been reported to be elevated in AAA tissue, combined with a decrease in their cystatin inhibitors. Dipeptidyl peptidase I is a lysosomal cysteine protease, which is of central importance, as it promotes the activation of granule-associated serine proteases, including neutrophil elastase, cathepsin G, and proteinase 3 (87). Neutrophil proteases may essentially degrade all types of matrix fibrillar proteins and thus promote AAA progression and ultimate wall rupture. The site of final adventitial rupture is characterized by a high level of protease expression (88) and a prominent enrichment of leukocytes and focal neovascularization (89). Of interest, Lindholt et al. have reported a protective effect of calcification in the evolution of AAA, probably explained by the greater resistance of calcified tissue to proteolysis (90).

Abdominal aortic aneurysm biomarkers are of great scientific interest, as a specific biomarker for prediction of AAA rupture is urgently needed. There are no AAA-specific laboratory markers; however, neutrophil-related factors may have future prospects. Ramos-Mozo et al. showed that plasma levels of neutrophil gelatinase-associated lipocalin are increased in AAA patients and correlate with AAA growth, reflecting the potential activation of both resident and circulating neutrophils (91). Despite the fact that AAA-related biomarkers have the limitation of not being disease specific due to a strong connection to general atherosclerosis, MMP-9 levels were found to have a significant correlation with AAA diagnosis (92). In addition to the marker potential, neutrophils (and PMN-related factors) constitute a therapeutic target in AAA patients. Doxycycline can directly inhibit MMP activity, and it effectively suppresses the development of elastase-induced AAAs in preclinical models (93). In clinical trials, preoperative doxycycline therapy improved the proteolytic balance in human AAA by reducing aortic wall neutrophil content (94). Doxycycline treatment resulted in a 2.5-fold decrease of MMP-9 protein levels (95). Lindeman et al. could show a strongly reduced PMN and cytotoxic T-cell content of the aortic wall after a 2-week doxycycline treatment of AAA patients indicating that the doxycycline-mediated effects are not restricted to neutrophils (96).

## Treatment Indications

Biodegradation of the abdominal aortic wall determines aneurysmal growth. Average growth rates of AAAs below 55 mm in size range from 2 to 3 mm per year. Larger AAAs are associated with higher growth rates (4). Most AAAs are asymptomatic, and the vast majority is detected occasionally during routine investigations. Risk factors for progression to rupture comprise hypertension, age, female sex, and persistent smoking (4). Finally, the life-threatening risk of rupture has to offset the operative mortality for aneurysm repair. For small fusiform AAAs (AAA diameter 30–39 mm), the 12 months risk of rupture is 0%, and it is still about 1% in those cases when the AAA diameter ranges between 40–49 mm (4). As a consequence, several studies recommended pursuing a surveillance policy in these cases (97). However, the 12 months risk of rupture rises exponentially with further increase of the aortal maximum diameter. Consequently, the threshold for aortic repair is 50 mm for women and 55 mm for men (4).

Besides the diameter of the AAA, its morphology plays another important role in the decision for repair. Fusiform AAAs are thought to be less prone to rupture than saccular AAAs, or those with eccentric components (Figure 1). Peak wall stress, presence of an ILT (Figure 2), and AAA wall mechanics are the factors most implicated with rupture risk. Therefore, early repair has been advised in these cases (98).

Symptomatic AAA is characterized by abdominal, back, or chest pain (99). Peripheral embolization with subsequent ischemia may be another sequel of AAAs with intraluminal thrombus (4). Peak wall stress is significantly greater in symptomatic or ruptured AAAs compared to asymptomatic AAAs according to a recent meta-analysis (98). As a consequence, early repair has been recommended in symptomatic patients (4, 98). Likewise, rapid aneurysm growth with more than 10 mm per year represents another indication for early repair (4). In conclusion, these recommendations aim to prevent AAA rupture, a life-threatening event with mortality rates of approximately 65–85% (100).

## Surgical Repair

**Figure 5:**
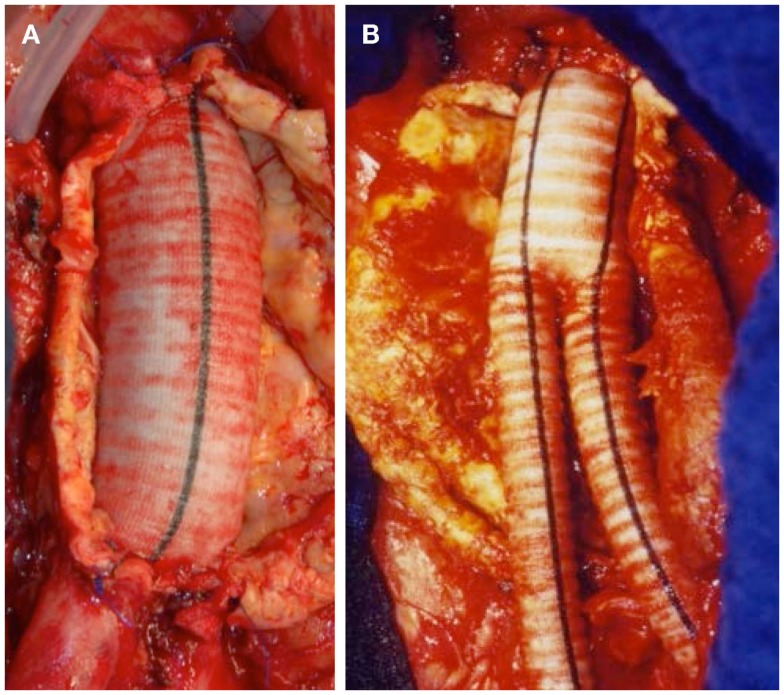
**(A)** Successful exclusion of an infrarenal AAA by a tube graft. Iliac bifurcation intact. **(B)** Successful exclusion of an infrarenal AAA by a bifurcated stent graft.

**Figure 6:**
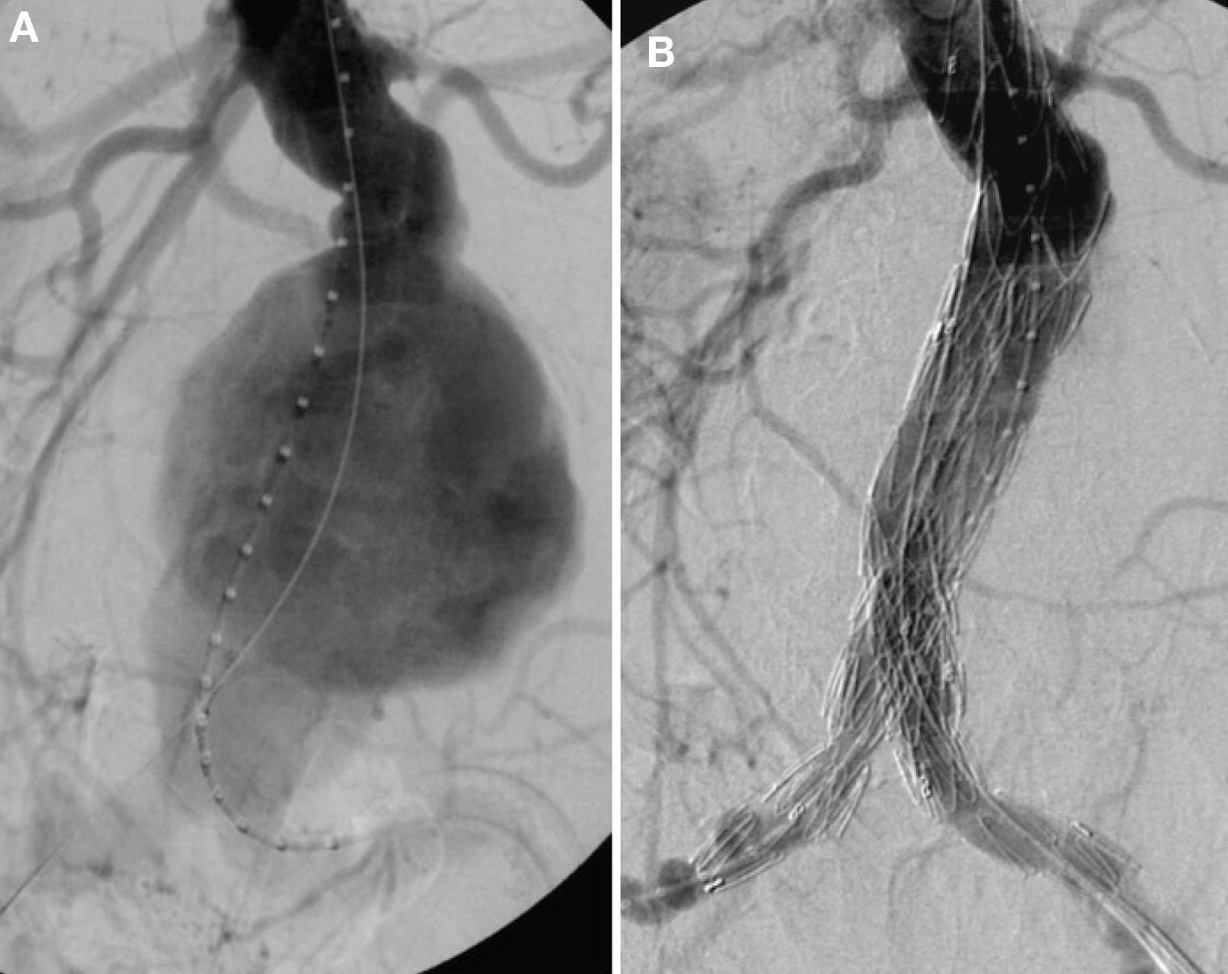
**(A)** Invasive angiography prior to endovascular repair (EVAR): rupture of an infrarenal AAA. **(B)** Final angiography after EVAR: successful exclusion of the ruptured AAA by a bifurcated stent graft; no endoleak visible.

Thorough preoperative evaluation of the patient’s comorbid diseases is a prerequisite for any type of surgical repair as its outcome essentially determines the decision between open or endovascular repair. Second, optimization of the treatment for various comorbidities should be obtained (4). Moreover, computed tomography (CT) or magnetic resonance angiograms are necessary to outline the morphological characteristics of AAAs for devising the operative strategies in open surgical repair (OSR) as well as for selection of the appropriate stent graft for endovascular aortic repair (EVAR). OSR is the mainstay in elective AAA cases. In general, tube grafts (Figure 5A) are preferred to bifurcated grafts, due to reduced dissection with less risk of injury to adjacent structures and consecutively shorter operating time (4). In case of additional iliac artery aneurysms or concomitant iliac arterial occlusive disease, indication for a bifurcated graft is given (Figure 5B). Since the introduction of EVAR via a transfemoral approach by Volodos in 1984 (101), this technique has become of wide-spread use (Figure 6).

An advantage of EVAR over OSR is less surgical trauma. Moreover, general anesthesia can be avoided. EVAR, however, requires adequate aortic and iliac fixation sites for effective sealing and fixation (4). Tortuous iliac arteries or extreme kinking of the aorta may prevent from adequate insertion or fixation of the stent graft in up to 40% of the patients. Endoleak after EVAR is a common complication in up to 25% of patients (4). In general, AAA patients suffer from a pro-thrombotic tendency. While the incidence of AAA-related disseminated intravascular coagulation (DIC) prior to surgery is rare and may be resolved upon AAA repair, the occurrence of DIC as a perioperative coagulopathy is a more frequent complication (102). In addition, a high incidence of venous thrombosis is observed after elective AAA repair despite systematic heparin application (103) and both coagulopathy as well as a hyperfibrinolysis are similarly encountered in ruptured AAA repair (104). It has been subject of investigation whether the two types of surgical intervention (OSR versus EVAR) differ in their hemostasis effects (27). Both were found to further aggravate the pro-coagulant and hyperfibrinolytic state of AAA patients in the initial post-operative period (105, 106) while reducing the circulating markers of deregulated hemostasis/fibrinolysis several months after AAA repair (107). Despite the fact that EVAR represents a smaller surgical trauma, a number of studies observed that EVAR as compared to open surgery led to higher marker levels in the immediate perioperative phase as well as the long-term period (108). This indicates that the inherent procedure and materials of EVAR may extend the hemostatic imbalance after AAA repair.

With respect to overall outcome, meta-analyses of prospective, randomized, controlled trials showed that 30-day mortality was higher in OSR (3.2–4.2%) versus EVAR (1.2–1.4%) in elective AAAs (109–111). However, there were no differences in the long-term (>4 years) all-cause mortality between EVAR (37.3%) and OSR (37.8%) (109, 110, 112). Causes of deaths were primarily cardiovascular events with similar incidences of cardiac death and fatal stroke, and malignant diseases (109, 112). Moreover, there were no significant differences in aneurysm-related mortality. Re-intervention rates, however, were significantly higher and more aortic related after EVAR (18.9%) compared to OSR (9.3%) (110).

The incidence of ruptured AAAs ranges between 6 and 18 per 100 000 person-years in Western countries (4). The overall mortality rate is extremely high with up to 80–90%. AAA rupture, defined as bleeding outside the adventitia of the dilated aortic wall, is classified into free rupture into the peritoneal cavity with extremely poor outcome or retroperitoneal rupture. Treatment strategies are of importance in aortic surgery, as clamping of the aorta is connected with a massive lower torso ischemia. Pre- (113) and post-conditioning (114) should activate the endogenous anti-oxidant defense mechanisms. Additionally, early infusion of radical scavengers plus L-arginine and cofactors (59, 61, 115, 116) play an important role to ameliorate deleterious consequences. Following these strategies, a reduction of 30-day mortality rates (52%) was noticed (117). In our own studies, the 90-day mortality rate for patients receiving OSR was 29% (118). In the last decade, a progressive increase in the proportion of patients managed by EVAR in case of ruptured AAAs was observed. Importantly, it has been shown that successful exclusion of ruptured AAAs by EVAR is feasible. Several studies showed significantly lower 30-day mortality rates after EVAR (24%) compared to OSR (52%) (111, 117). The survival advantage for EVAR after ruptured AAA persisted during the first 5 years after repair, but was lost after that period. The estimated 5-year survival was 44 and 39% for EVAR and OSR, respectively (117). By contrast, a recent meta-analysis including only randomized controlled trials failed to show superior outcome after EVAR compared to OSR (119). Moreover, long-term data are lacking for both survival and complications (119).

In conclusion, evaluation of the literature on OSR versus EVAR in both elective and ruptured AAAs failed to show superiority of one of these treatments in the long run, because randomized controlled trials do not consider the various risk factors, which account for the final outcome. Therefore, the tailored approach attributing geriatric patients with multiple morbidities to EVAR resulted in better outcome rates – at least initially (120, 121). In individuals considered unfit for OSR, no difference between EVAR and the non-intervention group with regard to all-cause mortality (21% in each group), with higher aneurysm-related deaths in the non-intervention group have been described (109). This finding may prompt us to avoid excessive surgery in geriatric and high-risk patients with multiple comorbidities. Future research, however, should aim at predictors for AAA growth and rupture. New biochemical markers along with functional imaging may help to select patients who are at risk at an early stage.

## Pharmacological Treatment Options

**Figure 7:**
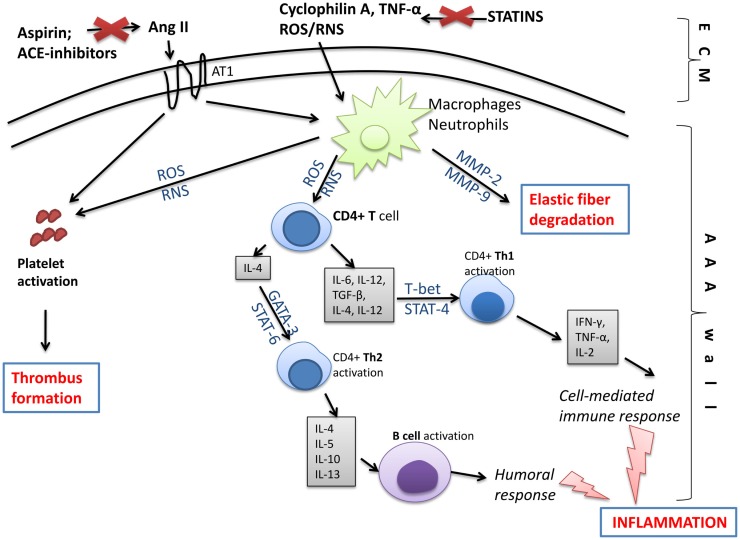
**Molecular changes in the aneurysmal wall, which may be targeted by pharmacological treatment**. Compounds generated in the extracellular matrix (ECM) like free oxygen or nitrogen species (ROS/RNS), inflammatory cytokines, e.g., tumor necrosis factor alpha (TNF-α) or cyclophilin A, and angiotensin II (through angiotensin receptor 1; AT1) stimulate inflammatory cells like macrophages or neutrophils, and platelets inside the AAA wall. Next, macrophages/neutrophils act on T and B lymphocytes initiating humoral and cell-mediated immune responses and leading to inflammation. As a consequence, pro-inflammatory signaling pathways involving NF-κB, T-bet, STAT-4, STAT-6, or GATA-3 are activated and a large amount of pro-inflammatory cytokines is released. Moreover, activated leukocytes produce matrix metalloproteinases (MMPs), which degrade elastic fibers inside the AAA wall. The activation of AT1 receptor and increased production of ROS/RNS by leukocytes activate platelets that form the intraluminal thrombus. Those changes, however, may be slowed down or eliminated by statins, anti-platelet drugs, or ACE inhibitors. ACE inhibitors, angiotensin-converting-enzyme inhibitors; Ang II, angiotensin II; AT1, angiotensin II receptor type 1; GATA, trans-acting T cell-specific transcription factor; MMP, matrix metalloproteinase; ROS, reactive oxygen species; RNS, reactive nitrogen species; STAT, signal transducer and activator of transcription; TGF-β, transforming growth factor beta; Th, T helper cell; TNF-α, tumor necrosis factor alpha.

Based on the outlined pathomechanism of disease, various pharmacological treatments are offered to AAA patients in addition to surgical intervention, which is limited to the progressed state (Figure 7). According to the European Society for Vascular Surgery guidelines for the management of AAA, statins and anti-platelet drugs should be used in patients diagnosed with AAA (4). Statins should be started 1 month before intervention to reduce cardiovascular morbidity and should be continued in the perioperative period for an indefinite duration; while aspirin at low doses should be prescribed on diagnosis and continued through the perioperative period unless a contradiction exists (4). However, only statins were indicated to both reduce cardiovascular mortality in AAA patients and to slow the rate of AAA growth. On the contrary, among the drugs that do not affect AAA growth but may be indicated for comorbidities are beta-blockers, angiotensin-converting enzyme (ACE) inhibitors, and AT1-receptor antagonists (122).

Abdominal aortic aneurysm is thought to be an inflammatory disease as patients with AAA exhibit increased values of inflammatory parameters such as C-reactive protein (123), or cytokines like tumor necrosis factor alpha (TNF-α) (77). Indeed, AAA and ILT are sources of physiologically active cells, including macrophages, T-cells, B-cells, and neutrophils that produce large amounts of messenger molecules (124). SMCs and endothelial cells in the AAA wall as well as red blood cells and platelets in the intraluminal thrombus are a source of free oxygen and nitrogen radicals. When activated platelets produce intracellular superoxide anion via NADPH oxidase, it conversely increases platelet recruitment favoring thrombus formation (125).

### Statins

The guidelines are based on experimental and clinical evidence of the positive effect of statins in AAA prevention and treatment. It was reported that statins may prevent aneurysm formation in animal models (126). Moreover, some observational studies in humans presented a 50% reduction in AAA expansion rate (127), and an association between statin therapy and a risk of AAA rupture (6). Also, a randomized trial reported that fluvastatin (80 mg daily for 30 days before surgery and continued until at least 30 days after surgery) halved both the primary 30-day outcome of post-operative myocardial ischemia and the secondary outcome of non-fatal myocardial infarction and cardiovascular death (128). A recent large meta-analysis showed that statins reduce the rate of progression of AAA (129). The meta-analysis by Galinanes et al. (130) reported that 1 week of statins administered to patients undergoing OSR or EVAR was associated with improved survival during 1 year after surgery and a decreased incidence of lower extremity embolic complications after EVAR.

In our previous report, we showed that simvastatin decreased the rate of free radical formation and the content of pro-inflammatory molecules like TNF-α in the aneurysmal wall (131). Therefore, protection of the AAA wall from ROS may be an important factor in the reduced AAA rupture risk. Furthermore, patients with a higher baseline C-reactive protein level respond better to statin therapy and have a lower absolute vascular risk than those without statins, as the findings of the JUPITER trial documented (132). The anti-inflammatory effect of statins may be in part connected with their influence on pro-inflammatory signaling pathways. Simvastatin taken for at least 6 months decreased the activity of NF-κB and ERK1/2 signaling pathways in the aortic wall of AAA patients (131, 133). A reduction in NF-κB activity under statins may be in part related with modulation of NF-κB in infiltrating T helper cells and CD40 signaling in SMCs and mononuclear cells (134), resulting in lower synthesis and release of IL-6 and IL-8 (135). Recently, van de Meij et al. (124) confirmed previous findings that patients who underwent AAA repair treated with simvastatin and atorvastatin at clinical doses had a reduced tissue content of macrophage-related markers and NF-κB dependent inflammatory molecules such as IL-6 and MCP-1, however, without a decrease in macrophage content.

Furthermore, inflammatory mediators regulate the expression and activity of MMPs released mainly by activated neutrophils and macrophages. In animal models, statin therapy suppressed the extent of AAA formation by 25%, and the incidence of AAA by 30% (136). The effect was associated with a reduction in MMP-9 protein and gene expression. In clinical trials, simvastatin was found to significantly lower MMP-9 concentrations by 40% within the aneurysm wall compared to placebo (137). Importantly, MMPs are inhibited when complexed with tissue inhibitors of MMPs (TIMPs). It was reported that MMP-9 and TIMP-1 as well as MMP-2 and TIMP-2 correlate with ILT thickness in patients with AAA (20). Therefore, statins may have beneficial effects to slow AAA growth.

### Anti-platelet therapy

Meta-analyses of randomized trials on primary and secondary prevention of AAA with anti-platelet therapy suggested that all patients diagnosed with AAA should be started on aspirin therapy at the time of AAA diagnosis as the use of low-dose aspirin may be associated with a reduction in all vascular deaths (138). Recent epidemiologic data indicate that the initiation of lifelong aspirin therapy should be considered as soon as a diagnosis of AAA is made (139). However, a retrospective study, which investigated aortic aneurysm cases between 1999 and 2006 from the National Health Insurance Research Database found no association between low-dose aspirin exposure and mortality or exacerbation in different types of aortic aneurysms (140).

Animal studies also showed that aspirin may significantly reduce both aortic plaque size and thrombus formation after vessel injury (141). Those effects may be in part mediated by the anti-oxidant effect of aspirin in atherosclerotic vessels (142). Aspirin treatment also leads to a reduction in free radical stress evident by decreased lipid peroxidation and significantly prevented reduction in glutathione content in endothelial cells of hypercholesterolemic animals (143). Therefore, based on animal models, anti-platelet therapy is expected to be beneficial to AAA patients.

### ACE inhibitors

Activation of the renin–angiotensin system has been implicated in the genesis of several cardiovascular disorders including AAA (144). Angiotensin II (Ang II) is strongly upregulated in human aortic aneurysms, and the Ang II increase is mediated by pathways dependent on ACE and chymase (145). In experimental studies, ACE inhibitors were found to reduce AAA rate (146), and in a retrospective clinical study, they were associated with a reduction in the risk of AAA rupture (147). However, there are controversial data. Some studies indicated that AAA patients treated with ACE inhibitors, but not those treated with other anti-hypertensives, seemed to be less likely to present with ruptured AAA as a recent Canadian study showed (147). By contrast, Wilmink et al. did not confirm the beneficial effect of ACE inhibitors on AAA progression (148), while others found a reverse, negative effect of ACE inhibitors on AAA (149).

Recently, Kortekaas et al. (150) presented results where patients treated with the ACE inhibitor ramipril (5 mg/day, for 4 weeks) had significantly lower levels of NF-κB and Ang II activity in AAA tissue and a lower content of IL-8 and MCP-1. The effect of ACE inhibitors on inflammatory mediators may result in a change of cell activation and, for instance, a shift in macrophage signature toward a predominance of alternatively activated macrophages. This may at least in part, account for the reduced expression of the proteases MMP-9, cathepsin L, and S, which are all considered instrumental in the process of AAA growth (85). Moreover, ACE inhibitors influence elastolytic MMP levels in the AAA wall to reduce elastin degradation within the vessel (146).

## Conclusion

Substantial progress has been made in recent years in understanding the process of AAA development and progression. The ILT has emerged as a major player, which “entraps” leukocytes, in particular neutrophils, to create a pro-oxidant and proteolytic environment that leads to vessel wall destabilization. The early processes of aneurysm development may be particularly sensitive to changes in the pathways controlling oxidative or nitroxidative stress where localized deregulation may induce endothelial and SMC dysfunction and promote thrombus formation. Thus, pharmacological treatment options for AAA patients progressively incorporate anti-oxidant, anti-inflammatory, and anti-proteolytic drug effects in addition to cholesterol, hemostasis, or blood pressure control. The need for surgical repair is carefully evaluated based on disease progression, morphological AAA characteristics, and patient comorbidities to avoid unnecessary risks. Predictive markers such as D-dimer for AAA growth are required to evaluate the risk for imminent rupture and further improve disease control.

## Conflict of Interest Statement

The authors declare that the research was conducted in the absence of any commercial or financial relationships that could be construed as a potential conflict of interest.
